# Leptin Inhibits Neutrophil Apoptosis in Children via ERK/NF-κB-Dependent Pathways

**DOI:** 10.1371/journal.pone.0055249

**Published:** 2013-01-31

**Authors:** Zhizhi Sun, Stéphane Dragon, Allan Becker, Abdelilah S. Gounni

**Affiliations:** 1 Department of Immunology, University of Manitoba, Winnipeg, Manitoba, Canada; 2 GREAT ICE (the Gender Related Evolution of Asthma Team Inter-disciplinary Capacity Enhancement), University of Manitoba, Winnipeg, Manitoba, Canada; National Institute of Infectious Diseases, Japan

## Abstract

**Introduction and Rationale:**

Previous studies have shown that delayed neutrophil apoptosis is associated with chronic airway diseases. Leptin is an adipocyte-derived hormone that acts as a regulator of energy homeostasis and food intake. Emerging evidence suggests that leptin can regulate immune responses including the release of proinflammatory cytokines and protection of inflammatory cells from apoptosis. Serum leptin is increased during allergic reactions in the airways. However, the expression and function of leptin receptor in neutrophils isolated from children is not known.

**Methods:**

Flow cytometry was used to detect leptin receptor expression in neutrophils isolated from allergic asthmatic (n = 14), allergic non asthmatic (n = 21), non allergic asthmatic (n = 7) and healthy children (n = 23); confocal laser scanning microscopy combined with immunofluorescence was performed to detect intracellular pool of leptin receptor; Annexin-V/PI staining and caspase 3 activity was used to determine neutrophil survival. Pharmacological inhibitors were utilized to understand the role of MAPK and NF-κB pathway in leptin-induced neutrophil survival.

**Results and Conclusion:**

A heterogeneous leptin receptor expression was observed on neutrophils isolated from children. Neutrophils isolated from healthy children expressed more leptin receptor than those from allergic asthmatic (P<0.05) but not allergic non-asthmatic (P>0.05) or non-allergic asthmatic children (n = 7, P>0.05). Neutrophils isolated from children express an intracellular pool of leptin receptor that was mobilized to the cell surface upon GM-CSF stimulation. Finally, leptin exhibited anti-apoptotic properties on neutrophils via NF-κB and MEK1/2 MAPK pathway. Collectively, our data suggest that leptin may enhance airway inflammation by promoting neutrophil survival.

## Introduction

Neutrophils are essential components of the innate immune system, and they form a primary defense against invading microorganisms [1]. Neutrophils are capable of releasing active mediators like TNF and IL-8/CXCL8 which may contribute to the pathogenesis of airway inflammatory diseases. Delayed neutrophil apoptosis is associated with inflammation in the airways [2]. A variety of pro-inflammatory cytokines can induce neutrophil anti-apoptotic or pro-survival effects including IL-1β, IL-4, IFN-γ, G-CSF, and GM-CSF [3], [4], [5], [6].

Leptin is an adipocyte-derived pleiotropic cytokine-like hormone that acts as a regulator of energy homeostasis and food intake. Plasma levels of leptin are positively correlated with adipocyte size and were found to be increased in obese patients [7], [8]. High levels of serum leptin lead to deregulation of energy balance. Although patients with high levels of serum leptin were shown to suffer from sepsis and other inflammatory diseases [9], [10], some reports suggest an immune-optimizing and pro-survival role of leptin in mouse models of sepsis [11]. Leptin exerts its biological functions by binding to short and long forms of leptin receptors (also called obesity receptors or ObR) which are generated by alternative splicing. A third type called secretory isoform of leptin receptor is also known [12], [13]. These receptors are expressed in a wide variety of cell types and tissue compartments including lung, kidney, adrenal gland, hematopoietic progenitors, and leucocytes [14]. The signaling pathways activated by leptin/ObR mimic the characteristics of both cytokine and growth factor/hormones [15], [16]. Leptin up-regulates macrophages/monocytes phagocytic function, and proinflammatory cytokine secretion [17]. Besides, it also modulates neutrophil chemotaxis and reactive oxygen species release [18], [19]. Leptin is also involved in NK cell development, differentiation, proliferation, activation, and cytotoxicity [20].

Human neutrophils express the receptors for a number of cytokines and chemokines such as IL-4, IL-8/CXCL8, IL-13, and GM-CSF. It has been reported that the short form of leptin receptor is expressed in peripheral blood neutrophils isolated from healthy adult subjects both *in vitro* and *in vivo*, and neutrophil apoptosis is inhibited by leptin *in vitro* [21]. Leptin has been shown to augment bacterial phagocytosis, intracellular hydrogen peroxide production and chemotactic migration of neutrophils [19], [22]. Interestingly, leptin was also shown to enhance the cytokine release, migration, and survival of eosinophils, collectively suggesting a role in allergic inflammation [23], [24]. To date, however, the expression and function of leptin on neutrophils purified from children in context of allergic airway disease has not been studied.

Here, we first demonstrate a heterogeneous leptin receptor expression on neutrophils isolated from children. Neutrophils isolated from healthy children (n = 23) expressed more leptin receptor than those from allergic asthmatic (n = 14, P<0.05) but not allergic non asthmatic (n = 21, P>0.05) or non allergic asthmatic children (n = 7, P>0.05). Interestingly, GM-CSF induced surface expression of leptin receptor on neutrophils children. Furthermore, in addition to p38 mitogen activated protein kinase (MAPK) and phosphatidylinositol 3-kinase (PI3K) pathways, leptin exhibited anti-apoptotic properties on neutrophils isolated from children via nuclear factor kappa B (NF-κB) and MEK1/2 MAPK pathway.

## Materials and Methods

### Reagents

Ready-made cycloheximide solution, Pyrrolidine Dithiocarbamate (PDTC), FITC-conjugated mouse IgG1 anti-human CD16/FcγRIII mAb, mouse IgG1 (clone MOPC-21) isotype control, and propidium iodide were obtained from Sigma (Sigma, Oakville, ON, Canada). FITC-conjugated annexin-V was purchased from BD Biosciences (Mississauga, ON, Canada). SB203580, U0126, and wortmannin were purchased from Calbiochem (San Diego, CA, USA). Anti-Fas IgM (CH11) was purchased from Upstate (MA, USA). Recombinant human GM-CSF was from PeproTech (Dollard des Ormeaux, QC, Canada). HyQ RPMI 1640 and fetal bovine serum (FBS) were obtained from Hyclone Laboratories (Logan, UT). Penicillin and streptomycin were purchased from Gibco BRL (Life Technologies Inc., Burlington, ON, Canada). Dextran and Ficoll-Paque histopaque were obtained from Amersham Pharmacia Biotech (GE Healthcare, Baie d'Urfe, QC, Canada). Wright-Giemsa stain was from Fischer Scientific (Ottawa, ON, Canada). Unless otherwise stated, all other reagents were purchased from Sigma.

### Isolation and purification of human peripheral blood neutrophils

This study was approved by the Research Ethics Committee of the Faculty of Medicine at the University of Manitoba. Parents of all the study participants (children) provided written informed consent. Blood was collected into sterile heparin tubes from the peripheral vein of allergic asthmatic, non allergic asthmatic, non asthmatic allergic, and healthy children (Table 1). Clinical assessment for asthma was performed by a pediatric allergist (A.B.) and was based on the Canadian Asthma Consensus Guidelines in which symptoms and variable airway obstruction were two major criteria [25]. To confirm the asthma diagnosis, pediatric allergist conducted a physical examination for chest symptoms (hyperinflation, wheeze, prolonged expiration and decreased breath sounds) and a standardized history, including questions on cough, wheeze, shortness of breath, response to current medications (i.e. bronchodilators, corticosteroids), and the presence of other allergic conditions (e.g. allergic rhinitis, atopic dermatitis and food allergies)

**Table 1:** Clinical characteristics of the children.

	Asthmatic non allergic	Allergic non asthmatic	Allergic asthmatic	Healthy control
Number of Patients	7	21	14	23
FEV1	1.73±0.086	1.78±0.060	1.71±0.076	1.82±0.075
PC20	9.02±3.63	10.01±2.63	8.72±4.73	13.15±4.27
CRP (ng/mL)	2045.69±1521.39	2763.89±1141.08	997.26±475.71	2807.42±790.23

All children were between the ages of 10–11years. FEV1; Forced expiratory volume in 1 second, PC20; provocative concentration causing a 20% fall in FEV1, CRP; C-reactive protein.

Neutrophils were separated from whole blood by 30 min Dextran-500 sedimentation at room temperature followed by Ficoll-Paque histopaque density gradient centrifugation [26]. After hypotonic lysis of residual erythrocytes, cells were washed twice with PBS and resuspended in RPMI 1640 medium supplemented with 10% heat-inactivated FBS, 100 U/ml penicillin, 100 µg/ml streptomycin and incubated at 37°C in 5% CO_2_. Cytological examination of stained neutrophils by the Wright-Giemsa method accounted for 95–98% cell purity (Table S1 and figure S1). Cell viability was greater than 98% as determined by trypan blue dye exclusion examination (Table S1).

### Cell culture

Neutrophils isolated from children were cultured at 1×10^6^ per ml in complete culture medium (RPMI 1640 containing 10% FBS) in the presence and absence of 10 ng/ml rGM-CSF, and 10 µg/mL leptin (R&D Systems, Minneapolis, MN, USA) for the indicated times. In some experiments, 1 µg/ml cycloheximide was also added. Blocking experiments were performed by using inhibitors SB203580 (25 µM), Wortmannin (10 µM), PDTC (5 µM), and U0126 (5 µM) in the culture as we previoulsy described [26], [27].

### Flow Cytometry analysis of leptin receptors expression on human neutrophils

Cell preparations of 2×10^5^ PMN in 100 µl of PBS were transferred into FACS tubes and were incubated for 45 minutes on ice with 12.5 µg/ml of primary antibodies mAb anti-leptinRor mouse IgG2A isotype control (R&D Systems). Cells were subsequently washed with PBS and incubated for 30 minutes in 1∶200 dilution of FITC-conjugated rat-anti mouse IgG (Jackson ImmunoResearch Laboratories, West-Grove, PA). To double check neutrophil purity, cell preparations were also stained with FITC conjugated anti-humanFcγRIII/CD16, marker of neutrophil, or isotype control MOPC21 (figure S1). Samples were washed again and analyzed on FACScan. Results are presented as geometric mean fluorescence intensity (MFI) or percentage of positive cell using Cell Quest software (Becton Dickenson, Oxnard, CA).

### Analysis of neutrophil apoptosis

Neutrophil apoptosis was detected by Annexin V/propidium iodide (PI) staining assay [26] to detect early apoptotic cells (having intact membrane with externalized phosphatidylserine (PS) residues), late apoptotic cells (apoptotic cells showing compromised membrane integrity), and non-apoptotic cells at 18 h of culture. Cells were washed in ice-cold PBS and resuspended in 100 ml of annexin V binding buffer (140 mmol/L NaCl, 2.5 mmol/L CaCl_2_, 1.5 mmol/L MgCl_2_, and 10 mmol/L HEPES, pH 7.4) containing annexin V-FITC and PI (1 µg/ml) for 15 minutes. FACS analysis was performed with CellQuest-Pro software, and cells negative for both annexin V and PI were considered viable (survival).

### Caspase 3/7 enzymatic activity assay

Caspace enzymatic activity was assessed using Caspase Glo kit. In brief, 15×10^4^ cultured neutrophils were subjected to a Caspase-Glo (Promega) substrate solution according to the manufacturer's instructions. Caspase enzymatic activity was acquired by an EG&G Berthold microplate luminometer after a 60 min incubation.

### Immunofluorescence (IF) and confocal laser scanning microscopy (CLSM)

Intracellular pool staining of leptin receptor was performed using IF and CLSM. In brief, 1×10^5^ fresh cells were displayed onto microscope slides by cytospin centrifugation (ThermoShandon, Pittsburg, PA) and fixed with 4% paraformaldehyde for 20 min at room temperature. Slides were washed three times with 0.05 M Tris-HCl buffered isotonic saline, pH 7.6 (TBS), air dried and stored at −20°C. Thawed cytopreparations were washed in TBS and subsequently treated for 20 min with a universal blocking solution (Dako Cytomation, Carpenteria, CA). Afterwards, slides were washed with TBS and incubated overnight at 4°C with anti-leptin receptor mAb or with the respective IgG2a isotype control (both at 10 µg/ml). After systematic washes, affinity purified rabbit anti mouse Alexa Fluor 488 F(ab)×2 fragment(10 µg/ml) was added sequentially for 1 h at room temperature. Slides were then extensively washed and counterstained for 5 min with propidium iodide (2 µg/ml). Following washes, slides were mounted with ProLong anti-fade agent, Molecular Probes (Life Technologies Inc.) and covered with a cover slip before being acquired under oil immersion at 100× magnification by confocal laser scanning microscopy (Olympus IX70 inverted microscope coupled to Fluoview confocal laser scanning system with a Cooke Sensicam) and analyzed with the Fluoview software (Mississauga, ON, Canada).

### Statistics

Differences between the groups were analyzed by using one-way ANOVA followed by Newman-Keuls multiple comparison test, and unpaired *t* test using GraphPad Prism v4.0 software. P values<0.05 were considered statistically significant

## Results

### Children neutrophils exhibit heterogeneous leptin receptor expression

**Figure 1:**
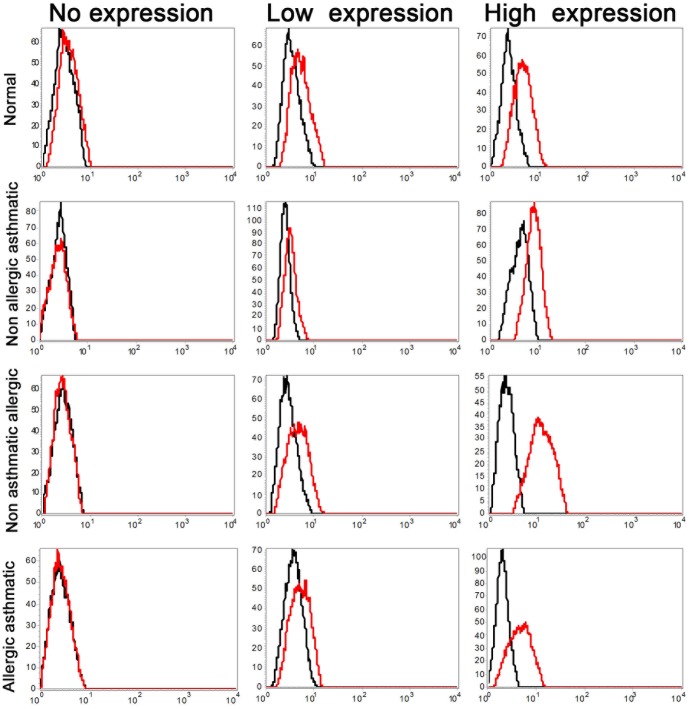
Cell surface expression of leptin receptor on peripheral blood PMNs. Highly purified peripheral blood children PMNs from allergic asthmatics, non-asthmatic allergic, non-allergic asthmatic, and normal controls were analyzed by FACS with mouse anti-human leptin mAb(red lines) and mouse IgG2a isotype-matched control Ab (black lines).

**Figure 2:**
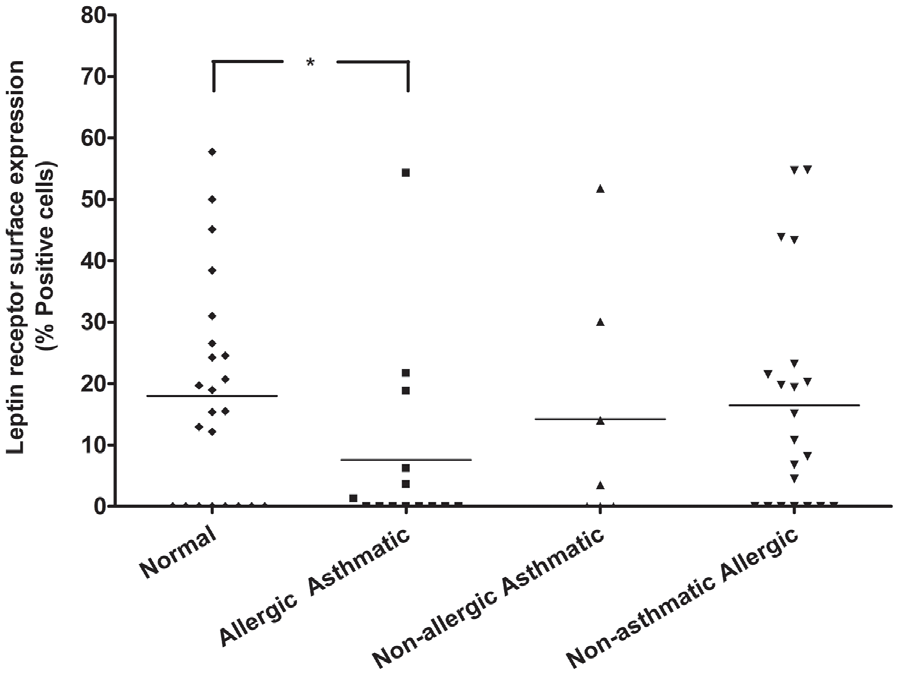
Comparative expression of leptin receptor in neutrophils of different groups of children. Summary of cumulative leptin receptor expression is shown. X axis represents different groups of children, classified as normal healthy, allergic asthmatic, non-allergic asthmatic, and non-asthmatic allergic children. Y axis shows percentage positive cells compared to isotype control. * p<0.05, unpaired *t* test.

To determine whether freshly isolated peripheral blood neutrophils from children express the leptin receptor, neutrophils were incubated with anti-leptin receptor mAb and analyzed by flow cytometry. The purity of human neutrophils was confirmed using CD16/FcγRIII staining as well as Wright-Giemsa staining (Figure S1, Table S1). Since anti-leptin receptor mAb binds to extracellular domain of both short and long forms of the leptin receptor, we detected any form of leptin receptor. As shown in Figure 1, heterogeneous expression of leptin receptor was detected in neutrophils isolated from asthmatic allergic (n = 14), non-asthmatic allergic (n = 21), non-allergic asthmatic (n = 7), and healthy children (n = 23). Figure 2 summarizes surface expression of leptin receptor in neutrophil isolated from all children. Neutrophil isolated from normal healthy children express more surface leptin receptor than neutrophil from allergic asthmatic children (P<0.05). Quantitatively, 36% (5 out of 14) of allergic asthmatic children and 65% (15 out of 23) of normal children express leptin receptor. In each groups of patients, leptin receptor was found to be absent, low expression, and high expression; as determined by percentage positive cells (0–2% = no expression, 3–15% = low expression, greater than 16% = high expression). Collectively, these results demonstrated that neutrophil isolated from children showed a heterogeneous expression of surface leptin receptor.

### GM-CSF upregulates the surface leptin receptor expression on neutrophil isolated from children

**Figure 3:**
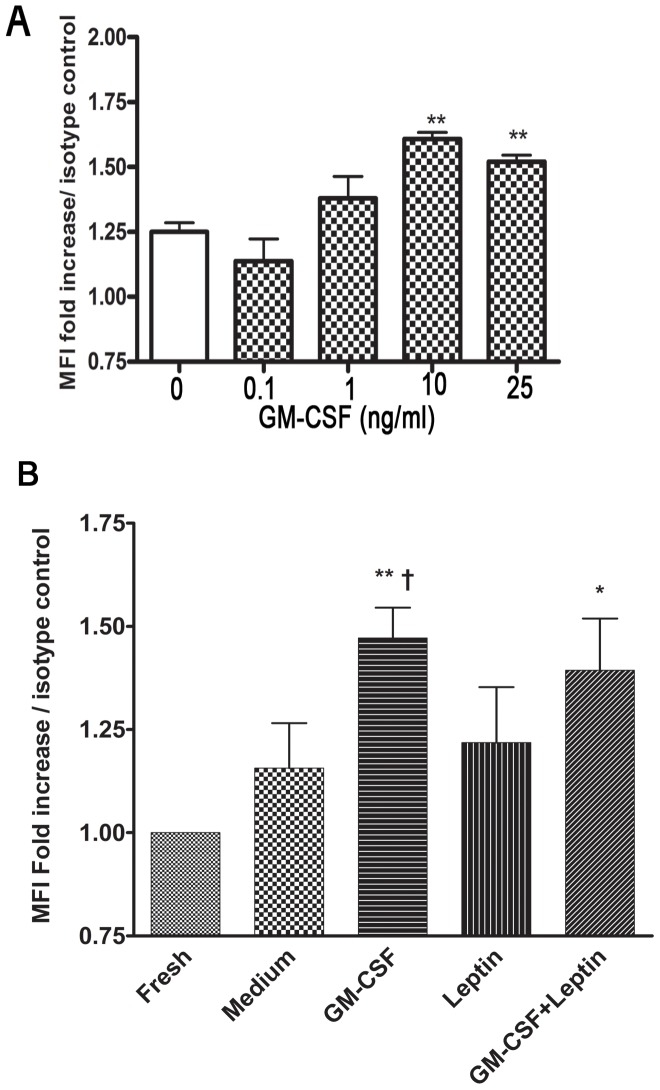
GM-CSF up regulates surface leptin receptor expression in peripheral neutrophils isolated from children. A. Dose response effect of GM-CSF (0.1, 1, 10, 25 ng/ml) on leptin receptor expression on neutrophils from children (n = 3, P<0.01). B. Neutrophils incubated with GM-CSF (10 ng/ml), leptin (10 µg/ml), combination, or alone for 2 h were analyzed by FACS with mouse anti-human leptin mAb and mouse IgG2a isotype-matched control Ab. Mean fluorescence intensity (MFI) of anti-leptin receptor is plotted as fold increase compared to isotype control. (n = 7, data shown as mean ± SD) **p<0.01, *p<0.05 compared to freshly isolated group, one-way ANOVA; †p<0.05 compared to medium control, unpaired *t* test.

To investigate the mechanisms underlying leptin receptor expression in neutrophils isolated from children, we tested whether leptin surface receptor can be upregulated upon GM-CSF stimulation. Compared to freshly isolated neutrophils at steady state, leptin receptor was found to be upregulated after stimulation for 2 hours with GM-CSF in dose dependent manner with the highest effect observed at 10 ng/ml (Figure 3A, n = 3, P<0.05).

Neutrophils isolated from 7 different children were then incubated with leptin (10 µg/ml), GM-CSF (10 ng/ml), or combination for 2 h followed by FACS analysis of leptin receptor. Leptin did not enhance the effect of GM-CSF and no significant differences were found between fresh cells in steady state, medium or leptin -stimulated stimulated conditions at 2 h (Figure 3B, P>0.05, n = 7). Our data suggest that neutrophil possess intracellular store of leptin receptor that is mobilized upon GM-CSF priming.

**Figure 4:**
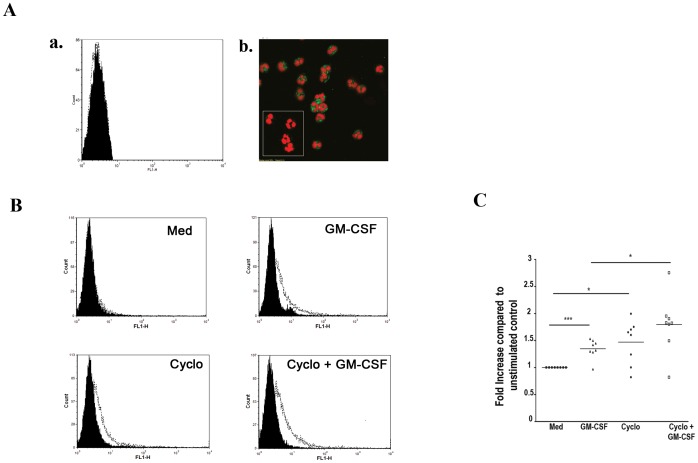
GM-CSF mobilizes the intracellular pool of leptin receptor to the cell surface. *A*.(a *and b*). Representative FACS data showing absence of surface leptin receptor expression in freshly isolated neutrophil. *b*. Immunofluorescence of leptin receptor on neutrophil (inset shows isotype control), also stained with PI for nuclei (red color). Intracellular pool of leptin receptor (green color) is observed from freshly purified neutrophils in which no expression of leptin receptor was observed by FACS. Isotype control antibodies were used as negative controls. *B–E*. Normalized neutrophil leptin receptor expression in fresh cells at 2 h in medium, GM-CSF, Cycloheximide (10 ng/ml), and GM-CSF+Cycloheximide, quantitated in *F*. *p<0.05, ***p<0.001 compared to unstimulated control, unpaired *t* test.

To further investigate this possibility, we first performed immunofluorescence analysis on freshly purified blood neutrophils that has no surface leptin receptor expression as detected by FACS (Figure 4A.a). Intracellular staining of leptin receptor using immunofluorescence demonstrated the existence of intracellular pool of leptin R protein in those human neutrophils isolated from children (Figure 4A.b). Furthermore, incubation of these cells with GM-CSF (10 ng/ml) for 2 h induces significant surface leptin receptor expression compared to unstimulated cells (Figure 4B, P<0.05, n = 7). Blocker of protein synthesis cycloheximide did not inhibit GM-CSF induced leptin receptor surface expression (Figure 4B and C, n = 7). Our results suggest the presence of intracellular pool of leptin receptor and priming effect of GM-CSF up-regulates leptin receptor surface expression on neutrophils purified from children.

### Leptin inhibits neutrophil apoptosis via NFκB activation in children

**Figure 5:**
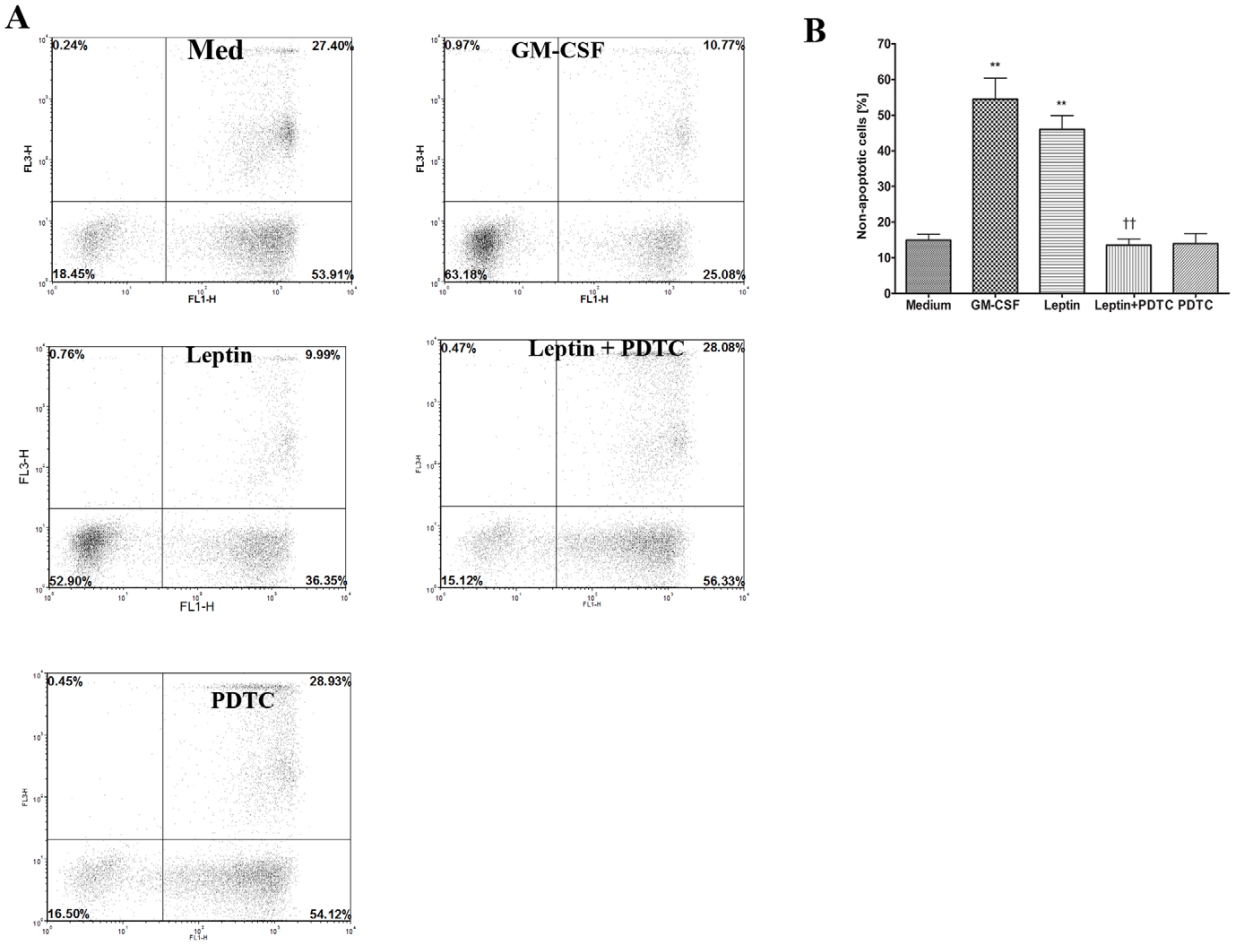
Leptin inhibits neutrophil apoptosis via NFκB involvement. ***A.*** Representative Annexin-V/PI staining assay of neutrophil apoptosis after 20 h incubation. ***B.*** Quantitative analysis of non-apoptotic cells in neutrophil from 5 children after 20 h incubation with untreated (medium), 10 ng/ml GM-CSF, 10 µg/ml Leptin, Leptin+PDTC (5 µM) and PDTC alone.n = 5 ** p<0.01 compared to medium alone, ††p<0.01 compared with leptin-stimulated group, one-way ANOVA.

Previous studies have shown that leptin can delay neutrophil apoptosis *in vivo* and *in vitro* with high concentration of leptin (0.5 µM) in adults [21]. To date, the effect of leptin on neutrophil survival in children is not determined. To test whether stimulation with leptin has the same effect on neutrophils isolated from children as in adults [21], purified blood neutrophils from children were treated with leptin and medium alone for 20 h. The neutrophil apoptosis was examined by Annexin V/PI staining assay by using flow cytometry; a representative analysis of 5 subjects is shown in Figure 5A. In summary, 18.43% survival in untreated medium, 52.90% survival in stimulation with 10 µg/ml leptin, and 63.16% survival in stimulation with 10 ng/ml GM-CSF were observed (Figure 5A). We also investigated the enzymatic activity of executioner caspase-3 in neutrophils from children treated with leptin, GM-CSF, or med. Caspase 3 enzymatic activity measured at 6 h revealed a 3-fold decrease in neutrophils treated with leptin compared with untreated cells (Figure S2, n = 3, p<0.001). Furthermore, reduced caspase-3 protease activity were maintained up to 24 h in leptin or GM-CSF treated neutrophils (Data not shown). It was clearly noticeable that leptin itself has an effect in enhancing the neutrophil survival from children (Figure 5B, n = 5, P<0.05 compared to medium alone).

To study the signaling pathways of leptin-induced neutrophil survival, we employed PDTC, a competitive inhibitor of NFκB pathway. As shown in figure 5A, and B, survival response of up to 16.50% in cells incubated with PDTC (5 µM) and leptin (10 µg/ml), and 15.12% in PDTC alone was observed (n = 5, P<0.05).

**Figure 6:**
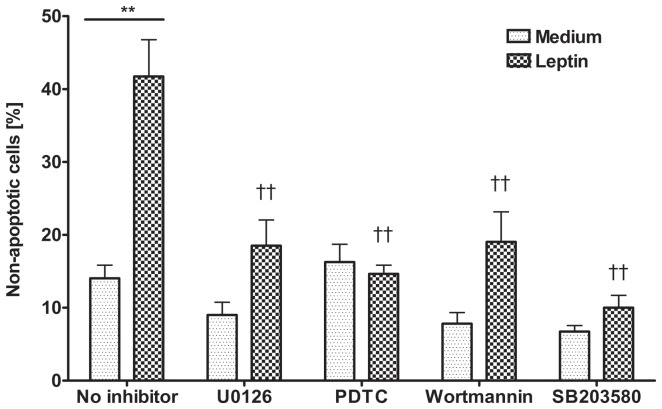
Pharmacological inhibition of MEK1/2 MAPK and NFκB blocks anti-death effects of leptin in neutrophils from children. Neutrophils were cultured in the presence and absence of leptin (10 µg/ml) for 20 h (n = 3). U0126 (5 µM) significantly prevented the leptin-mediated anti-death effect while PDTC (5 µM) completely blocked anti-death effect. SB203580 (p38 inhibitor, 25 µM) and Wortmannin (PI3K inhibitor, 10 µM). **p<0.01 compared to medium control, ††p<0.01 compared to leptin-stimulated group without any inhibitor; one-way ANOVA.

Furthermore, we employed U0126 (an MEK1/2 MAPK inhibitor) to assess the role of this pathway in neutrophil survival. The percentage of non-apoptotic cells in media and inhibitors with/without leptin stimulation are summarized in Figure 6. Both inhibitors (5 µM) accelerated spontaneous neutrophil apoptosis but not significantly compared to unstimulated cells. U0126 significantly, but partially, prevented the leptin-mediated anti-apoptotic effect while PDTC totally blocked this effect (p<0.01, n = 3). The use of other MAPK inhibitors such as p38 (SB203580, 25 µM) and PI3K (Wortmannin, 10 µM) also inhibited significantly the anti-apoptotic effect of leptin on neutrophils isolated from children, as shown in previous studies in neutrophils from adult [21]. Our findings therefore show that leptin exhibits anti-apoptotic effects on children neutrophils via NFκB and MEK1/2 MAPK pathway, besides the previously studied p38 MAPK and PI3K pathways.

## Discussion

In this study, we demonstrated that the leptin receptor expression on the surface of peripheral blood neutrophils from children is heterogeneous. Neutrophils from healthy children express more leptin receptor than neutrophils from asthmatic allergic children. GM-CSF is able to prime neutrophils and up-regulate leptin receptor via mobilization of the intracellular pool to the cell surface. Leptin shows anti-apoptotic properties on neutrophil from children via NFκB and MEK1/2 MAPK pathway. Our findings suggest a role of leptin in enhancing neutrophil survival in asthma disease which are associated with altered levels of leptin receptor (27).

We observed that the neutrophils from normal children express more leptin receptor than those from asthmatic allergic children. The proportion of children with leptin receptor positive neutrophils is preponderant in normal children than asthmatic allergic children. The underlying mechanisms of decreased leptin receptor in asthmatic allergic children neutrophils in our study are unknown. Interestingly, the leptin levels in these patients did not exhibit any significant correlation across different disease spectrum. However, our data corroborates a recent study wherein authors observed that a decreased airway epithelial leptin/leptin R expression is associated with adult human asthma severity and airway remodeling features such as subepithelial thickness and transforming growth factor (TGF)-β [28]. Interestingly, the presence of leptin has been suggested to be necessary for the induction and maintenance of the pro-inflammatory Th1 immune response [29], [30], the response contrary to Th2 that is observed in allergic asthma. Leptin mediates the release of pro-inflammatory cytokines, and previous [21] and current study suggests that it can delay spontaneous neutrophil apoptosis. Moreover, leptin contributes to neutrophil accumulation at inflammatory sites [31]. Previous studies have shown that chronic IL-1, IL-6, or TNF stimulation results in reduction of leptin levels in adipose tissue cultures [32], [33]. These observations might also explain down regulated leptin receptor expression in neutrophils from allergic asthmatic children.

Surface leptin receptor was enhanced by pro-inflammatory cytokine GM-CSF in our study. Furthermore, independently of disease status, neutrophil from children displayed an intracellular pool of leptin receptor that can be mobilized from cytosol to cell surface by GM-CSF. Since inflammation in the airways involves pro-inflammatory cytokine production including GM-CSF, and GM-CSF induces upregulation of leptin receptor, it is conceivable that leptin will have stronger signaling towards neutrophil infiltrate in the lung. Collectively, although it is uncertain whether attenuated leptin receptor expression in neutrophils from allergic asthmatic children is a cause or consequence; the current study suggest a plausible link between leptin, immune cells, and metabolic state which may underlie the debatable relationship between asthma and obesity [34], [35].

Leptin has recently emerged as a strong pro-survival factor in immune system. It has been shown to augment the proliferation of naïve CD4+ T cells [29]. Leptin has been shown to prevent the apoptosis of eosinophils [23], neutrophils from human adult [21], T lymphocytes [36], [37], monocytes [38], [39], NK cells [40], and other hematopoietic cells [41], neuroblastoma cells [42], and hepatic stellate cells [43]. More recently, leptin was shown to induce survival, migration, degranulation, and Th2 cytokine synthesis of human basophils, suggesting its emerging immunoregulatory role in allergic reactions [44]. The leptin-induced survival benefit conferred on neutrophils from children in our study confirms the previously observed pro-survival role in neutrophils from adult and most importantly, provides additional information in context of allergic inflammation. Leptin expression is increased in allergic asthmatic airways, and seems play a role in the relationship between asthma and obesity [28], [45].

Collectively, the increased leptin levels in airway allergic inflammatory response, subdued leptin receptor on allergic asthmatic children compared to healthy controls, and leptin-induced neutrophil survival suggest that the effect of leptin/leptin R in this heterogeneous disease process (asthma) is complicated and may partly be responsible for the neutrophilic inflammation observed in some of the asthma cases [28], [46]. Interestingly, attenuated leptin/leptin R expression has also been observed in smokers and patients of chronic obstructive pulmonary disease (COPD), a neutrophil-dominated disease [47].

It has been reported that the anti-apoptotic effects of leptin were concentration dependent and blocked by an anti-leptin receptor mAb. The efficacy of leptin to block neutrophil apoptosis was similar to G-CSF [21]. The current study also shows that leptin can inhibit neutrophil apoptosis in children. The efficacy of leptin to block apoptosis of neutrophil isolated from children was not as high as GM-CSF or G-CSF (data not shown) but it was significantly effective. Both p38 MAPK [48], [49], [50] and PI3K [51], [52] activation have previously been shown to mediate neutrophil anti-apoptosis. In addition, we found NFκB pathway and MEK1/2 MAPK as other essential pathways that leptin utilizes to inhibit apoptosis. Our findings are also consistent with previous finding where leptin induces nitric oxide synthase type II in C6 glioma cells via NFκB [53]. It also has been shown that leptin can stimulate the release of proinflammatory cytokines such as IL-1β, IL-6, and TNFα from human placenta and maternal adipose tissue via NFκB and inhibited by MEK1/2 MAPK inhibitor U0126 [54]. Leptin promotes differentiation and survival of human dendritic cells [30]. The reason for these responses in different cell types via NFκB is unclear; however NFκB could be major pathway of leptin peripheral effects. Although leptin has been shown to activate multiple signaling pathways such as MAPK, PI3K, Akt, JAK-STAT including STAT1, STAT3, STAT5 [37], [55], [56], key events in leptin-mediated cell survival are incompletely understood. In neutrophils, our study suggests the requirement of MEK1/2 and NF-κB pathways besides previously studied p38 and PI3K activation in leptin-induced neutrophil survival. NF-κB is a ubiquitously expressed transcription factor known to mediate several biological processes such as cell survival, migration, and proliferation through the expression of various genes including cytokines, adhesion molecules, chemokines, and growth factors. Although NF-κB was also shown to mediate leptin-induced CD40 expression on murine dendritic cells [56], it was important in leptin-induced cell survival as in dendritic cells [57]. Whether MEK1/2, p38 MAPK, PI3K and NF-κB pathways cross-talk in mediating leptin effects on neutrophils remain yet to be determined, however it is apparent that leptin can activate multiple signaling pathways in neutrophils besides its effects on other immune cells in activating them [58].

In summary, neutrophils from allergic asthmatic children express less leptin receptor compared with healthy controls, GM-CSF primes the pre-stored pools of leptin receptor in neutrophils, and leptin promotes neutrophil survival via MEK1/2, NF-κB, p38 MAPK, and PI3K pathways. Further studies are warranted to understand the complex yet critically important relationship between serum/plasma leptin levels, leptin receptor expression, and severity of allergic asthma.

## Supporting Information

Figure S1 **Purified neutrophils from children (A) express high level of FcγRIII/CD16 (B).** Neutrophils were purified as described in material and methods and analyzed by flow cytometry using side scatter/forward scatter, mAb against neutrophil marker CD16/FcγRIII. The same neutrophil preparations were stained by Wright Giemsa (C).
(TIF)

Figure S2 **Leptin suppresses Caspase 3/7 activity in neutrophils from children.** Enzymatic activity of caspase-3 was measured in neutrophils purified from children cultured in the presence of leptin (10 µg/ml), GM-CSF (10 ng/ml), or anti-Fas IgM (250 ng/ml) served as positive control. Data are reported as relative light unit (RLU) (n = 3, * p<0.001 compared to medium treated cells).
(TIF)

Table S1 **Purity and viability of peripheral blood neutrophils isolated from healthy, allergic asthmatic, non allergic asthmatic and non asthmatic allergic children.** Purity and viability were determined by Wright-Giemsa staining and trypan blue exclusion, respectively.
(DOCX)
