# Supplementary figures and images for: Leptin Inhibits Neutrophil Apoptosis in Children via ERK/NF-κB-Dependent Pathways
Source: PLoS One. 2013 Jan 31;8(1):e55249. doi: 10.1371/journal.pone.0055249 (PMC3561393; doi:10.1371/journal.pone.0055249)

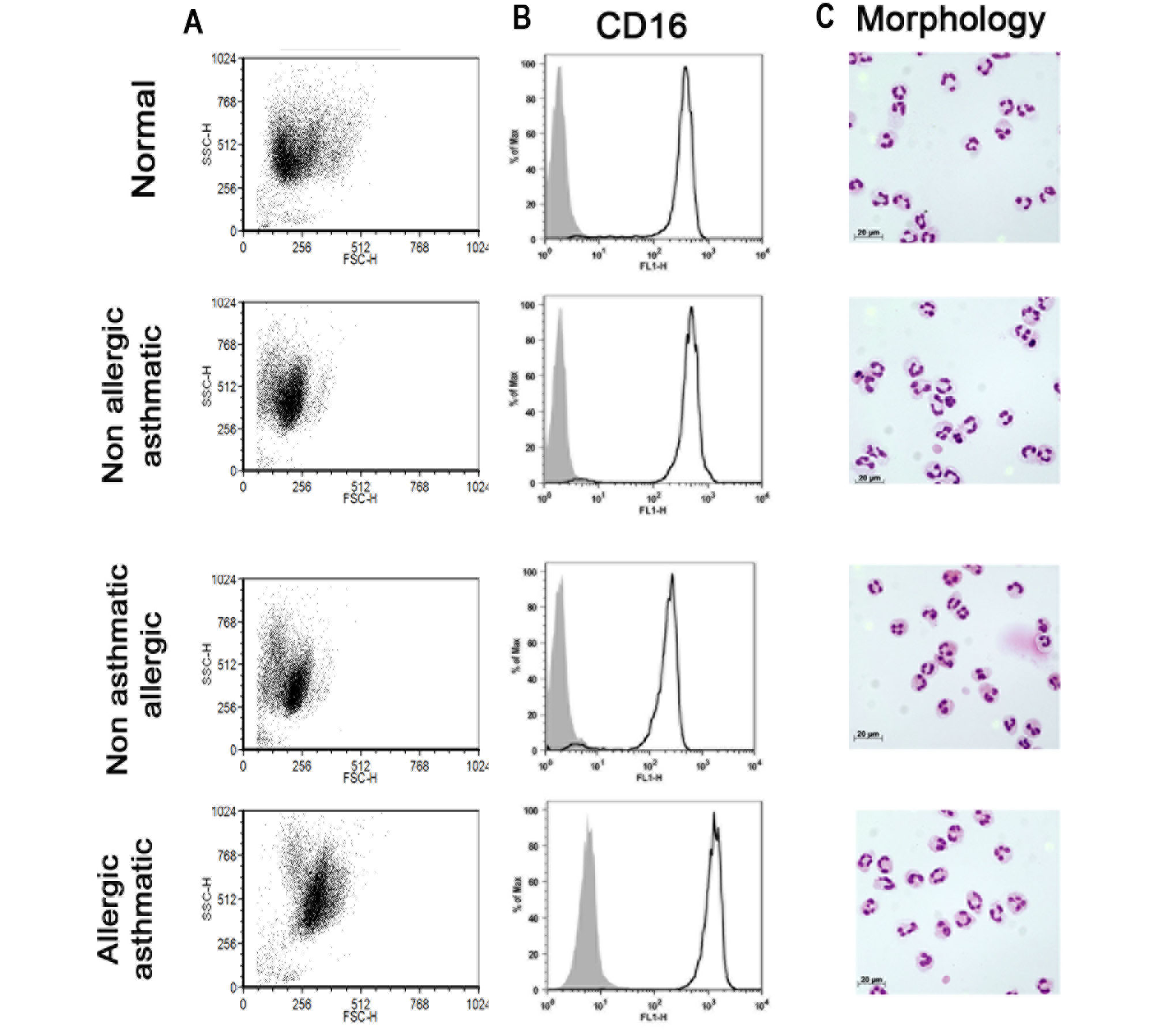

Supplement: Figure S1 — Purified neutrophils from children (A) express high level of FcγRIII/CD16 (B). Neutrophils were purified as described in material and methods and analyzed by flow cytometry using side scatter/forward scatter, mAb against neutrophil marker CD16/FcγRIII. The same neutrophil preparations were stained by Wright Giemsa (C). (TIF) [file pone.0055249.s001.tif]

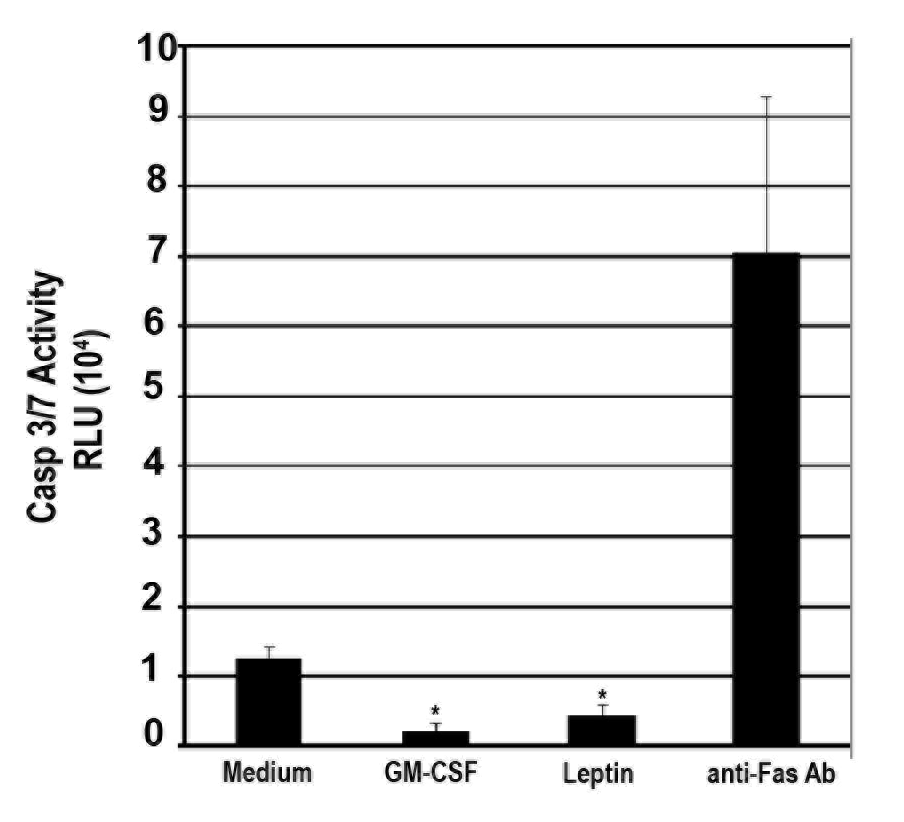

Supplement: Figure S2 — Leptin suppresses Caspase 3/7 activity in neutrophils from children. Enzymatic activity of caspase-3 was measured in neutrophils purified from children cultured in the presence of leptin (10 µg/ml), GM-CSF (10 ng/ml), or anti-Fas IgM (250 ng/ml) served as positive control. Data are reported as relative light unit (RLU) (n = 3, * p<0.001 compared to medium treated cells). (TIF) [file pone.0055249.s002.tif]
