# Supplementary material for: Leptin Inhibits Neutrophil Apoptosis in Children via ERK/NF-κB-Dependent Pathways
Source: PLoS One. 2013 Jan 31;8(1):e55249. doi: 10.1371/journal.pone.0055249 (PMC3561393; doi:10.1371/journal.pone.0055249)
Supplement: Table S1 — Purity and viability of peripheral blood neutrophils isolated from healthy, allergic asthmatic, non allergic asthmatic and non asthmatic allergic children. Purity and viability were determined by Wright-Giemsa staining and trypan blue exclusion, respectively. (DOCX) [file pone.0055249.s003.docx]

**Table S1: Purity and viability of peripheral blood neutrophils isolated from healthy, allergic asthmatic, non allergic asthmatic and non asthmatic allergic children.**

| Healthy | Cell Viability (%) | Purity (%) | Allergic Asthmatic | Cell Viability (%) | Purity (%) | Non-allergic Asthmatic | Cell Viability (%) | Purity (%) | Non-asthmatic Allergic | Cell Viability (%) | | Purity (%) |
| --- | --- | --- | --- | --- | --- | --- | --- | --- | --- | --- | --- | --- |
| #1 | 98 | 95 | #24 | 98 | 98 | #38 | 99 | 95 | #45 | 99 | 94 | |
| #2 | 99 | 97 | #25 | 99 | 97 | #39 | 97 | 93 | #46 | 98 | 98 | |
| #3 | 98 | 96 | #26 | 99 | 95 | #40 | 98 | 99 | #47 | 98 | 92 | |
| #4 | 98 | 99 | #27 | 99 | 96 | #41 | 96 | 97 | #48 | 99 | 94 | |
| #5 | 96 | 98 | #28 | 97 | 94 | #42 | 99 | 95 | #49 | 98 | 95 | |
| #6 | 96 | 91 | #29 | 98 | 93 | #43 | 98 | 96 | #50 | 97 | 96 | |
| #7 | 97 | 94 | #30 | 97 | 94 | #44 | 99 | 95 | #51 | 98 | 95 | |
| #8 | 99 | 95 | #31 | 99 | 98 |  |  |  | #52 | 99 | 99 | |
| #9 | 98 | 95 | #32 | 95 | 95 |  |  |  | #53 | 98 | 96 | |
| #10 | 99 | 97 | #33 | 99 | 96 |  |  |  | #54 | 97 | 94 | |
| #11 | 99 | 99 | #34 | 99 | 99 |  |  |  | #55 | 96 | 93 | |
| #12 | 99 | 95 | #35 | 99 | 92 |  |  |  | #56 | 99 | 95 | |
| #13 | 97 | 94 | #36 | 98 | 97 |  |  |  | #57 | 99 | 92 | |
| #14 | 98 | 99 | #37 | 97 | 95 |  |  |  | #58 | 98 | 99 | |
| #15 | 99 | 94 |  |  |  |  |  |  | #59 | 98 | 95 | |
| #16 | 96 | 92 |  |  |  |  |  |  | #60 | 97 | 92 | |
| #17 | 98 | 92 |  |  |  |  |  |  | #61 | 99 | 93 | |
| #18 | 99 | 92 |  |  |  |  |  |  | #62 | 99 | 91 | |
| #19 | 99 | 93 |  |  |  |  |  |  | #63 | 98 | 94 | |
| #20 | 99 | 97 |  |  |  |  |  |  | #64 | 99 | 95 | |
| #21 | 98 | 95 |  |  |  |  |  |  | #65 | 96 | 99 | |
| #22 | 98 | 94 |  |  |  |  |  |  |  |  |  | |
| #23 | 99 | 99 |  |  |  |  |  |  |  |  |  | |
| **Total** | **98.1±0.2** | **95.3±0.5** |  | **98.1±0.3** | **95.6±0.5** |  | **98±0.4** | **95.7±0.7** |  | **98±0.2** | **94.8±0.5** | |
